# Poor Sleep in Community-Dwelling Polysubstance Users: Association With Khat Dependence, Metacognition, and Socio-Demographic Factors

**DOI:** 10.3389/fpsyt.2022.792460

**Published:** 2022-05-10

**Authors:** Md Dilshad Manzar, Ahmad H. Alghadir, Masood Khan, Mohammed Salahuddin, Hamid Yimam Hassen, Ahmed M. Almansour, Dejen Nureye, Eyob Tekalign, Showkat Ahmad Shah, Seithikurippu R. Pandi-Perumal, Ahmed S. Bahammam

**Affiliations:** ^1^Department of Nursing, College of Applied Medical Sciences, Majmaah University, Al Majmaah, Saudi Arabia; ^2^Department of Rehabilitation Sciences, College of Applied Medical Sciences, King Saud University, Riyadh, Saudi Arabia; ^3^Department of Pharmacy, College of Medicine and Health Sciences, Mizan-Tepi University (Mizan), Mizan-Aman, Ethiopia; ^4^Pharmacology Division, Department of BioMolecular Sciences, University of Mississippi, Oxford, MS, United States; ^5^Department of Public Health, College of Medicine and Health Sciences, Mizan-Tepi University, Mizan-Aman, Ethiopia; ^6^Department of Medical Laboratory Sciences, College of Medicine and Health Sciences, Mizan-Tepi University, Mizan-Aman, Ethiopia; ^7^Department of Economics, College of Business and Economics, Mizan-Tepi University (Mizan), Mizan-Aman, Ethiopia; ^8^Somnogen Canada Inc., Toronto, ON, Canada; ^9^Saveetha Medical College and Hospitals, Saveetha Institute of Medical and Technical Sciences, Saveetha University, Chennai, India; ^10^The University Sleep Disorders Center, College of Medicine, King Saud University, Riyadh, Saudi Arabia; ^11^National Plan for Science and Technology, College of Medicine, King Saud University, Riyadh, Saudi Arabia

**Keywords:** polydrug use, khat, alcohol, nicotine, sleep problems, metacognition

## Abstract

**Purpose:**

Poor sleep and cognitive deficits are often associated with increased drug use. However, no study has addressed the relationship between poor sleep, substance dependence, and metacognitive deficit in polysubstance users.

**Methods:**

This was a cross-sectional study with a simple random sampling involving community-dwelling polysubstance users (*n* = 326, age = 18–43 years) in Mizan, Ethiopia. Participants completed a brief sleep questionnaire, severity of dependence on khat (SDS-Khat), a brief meta-cognition questionnaire, and a socio-demographic survey.

**Results:**

Majority (56.4%) of the polysubstance users had sleep disturbance. Chronic health conditions [adjusted odds ratio (AOR) = 2.52, 95% confidence interval (CI) 1.31–4.85], chronic conditions in the family (AOR = 2.69, 95% CI 1.40–5.20), illiterate-primary level of educational status (AOR = 2.40, 95% CI 1.30–4.04), higher SDS-Khat score (AOR = 1.39, 95% CI 1.13–1.72), and lower meta-cognition score (AOR = 0.90, 95% CI 0.84–0.97) predicted poor sleep in the polysubstance users. Moreover, low metacognition score and high SDS score also predicted additional sleep disturbances like chronic sleep insufficiency, lethargy and restlessness after nighttime sleep, socio-occupational dysfunctions, and daytime disturbances in polysubstance users.

**Conclusion:**

Poor sleep, severe khat dependence, and metacognitive deficits are common in community polysubstance users. Moreover, poor sleep is associated with higher khat dependence, lower metacognitive ability, lower educational status, and the presence of chronic conditions in polysubstance users or their families.

## Introduction

Polysubstance use (PSU) implies using more than one drug of abuse either simultaneously or sequentially within a defined timeframe. PSU is common among illicit drug users with the desire to (1) obtain greater effects compared to use of either drug alone, (2) acquire a notable increase in the subjective response to a drug, or (3) alleviate the adverse side effects of one substance by the other (1). Most substance use research (including preclinical research) has not accounted for PSU as a variable.

Globally, sleep disturbances have become one of the commonly prevalent mental health disorders, wherein one-third of general adult individuals suffer from sleep problems (2). Estimates vary, but a large proportion of community-dwelling adults show sleep disturbances ranging from ~16 to 65.4% (3–5). Poor sleep and related sleep disturbances may lead to physiological, psychological, and social disturbances (6). For example, individuals with poor sleep have been associated with treatment-resistant hypertension (7), suicidal ideation (8), dysregulated circulating cholesterol and triglyceride levels (9), and diabetes mellitus (10). Moreover, individuals with poor sleep quality were more susceptible to neuropsychiatric complications, especially substance use and affective and cognitive disorders (11–15). Intriguingly, the relationship between sleep disturbances and substance use disorders may be bidirectional, wherein sleep disturbances increase the risk of substance misuse (11, 12), and substance use may trigger sleep complications (16).

Substance-using populations in Ethiopian demographics have pronounced sleep problems (16–20). Habitual khat (*Catha edulis*) is a plausible explanation for changes in sleep patterns. Khat has two important alkaloids, cathinone and cathine, that possess stimulant-like activity similar to amphetamines (17, 21, 22). Central nervous complications are associated with khat use, including deficits in memory, concentration, sleep, headache, migraine, motor coordination, and stereotypical behavior (21, 22). Alcohol is often concurrently misused in the Ethiopian population (17, 18). The effect of alcohol on sleep continuity is dose-dependent, with low dose increasing the sleep time and high dose leading to short-term withdrawal state, increased sympathetic activity, and sleep disruption, mainly in the second phase of the night (23). Smoking tobacco is associated with a constellation of sleep complications, including difficulty initiating sleep, staying asleep, daytime sleepiness, and affective dysregulation, including anxiety and depression (24, 25).

Metacognitive abilities do vary among insomnia patients in comparison to healthy people (26). Some of the identifiable features of circadian rhythm (a component in sleep regulation) are associated with dysfunctional metacognition and neuroticism (27). Sleep quality characteristics, and metacognition mediate between chorotype measures and poor well-being (27). These pieces of evidence do imply that PSU disorder is a comorbid disorder commonly associated with numerous influences such as poor sleep, stress, and other factors. However, no study has attempted to assess sleep, sleep-related symptoms, and their predictors in community-dwelling polysubstance users. There is a paucity of studies regarding these factors among polysubstance users. Therefore, this study explored the prevalence of poor sleep, poor sleep-related symptoms, severe dependence on khat, and level of metacognitive deficits in polysubstance-using community adults. We hypothesized that history of chronic conditions in polysubstance users/family members, lower metacognitive ability, and higher level of dependence on substance use may predict poor sleep outcome.

## Materials and Methods

### Participants and Procedure

A cross-sectional study was performed on community-dwelling habitual polysubstance users living in Mizan-Aman, Bench Maji Zone, Ethiopia. Houses were earmarked using simple random sampling (lottery method) from the list of houses provided by health post professionals. All households with a minimum of one adult member were the source population. Adults with habitual use of more than one substance for at least 6 months composed the study population. PSU was defined as the habitual use of two or more of these substances: khat, alcohol, smoking, and caffeinated drinks. Those having memory problems or on neuro-psychotic medications based on self-report or on account of information given by the family members were excluded to avoid memory-related bias. A final sample (*n* = 326) with certain age (range: 18–43 years; mean: 27.1 ± 3.7 years) completed this study involving a brief sleep questionnaire (BSQ), severity of dependence on khat (SDS-Khat), a brief metacognition questionnaire, and a socio-demographics tool (19, 28). A brief and precise summary of the objectives and methods to be followed in the study were given to the participants. Participation was voluntary and involved no risks or rewards. The participants gave informed written consent for participation and publication.

### Measures

#### Brief Sleep Questionnaire

A BSQ with four dichotomous items (yes/no) was used to assess the presence of poor sleep and poor sleep-related symptoms. The BSQ items recorded responses to determine these: (i) subjective report of sleep disturbances; (ii) duration of sleep complaints (3 or more months); (iii) daytime restlessness, irritability, and tiredness; and (iv) report of social and occupational disruptions related to sleep disturbances. The respondents were identified as having poor sleep if they had any one of the first two symptoms, i.e., (i) or (ii) along with complaints of both (iii) and (iv) (20). Similarly, a clinical interview using slightly modified criteria based on the International Classification of Sleep Disorders, Revised (ICSD-R) has been used in previous sleep research in similar settings (4, 29). The BSQ was found to have an excellent level of internal consistency, as shown by a McDonald's Omega of 0.88 and the greatest lower bound to reliability of 0.92 in this study sample (30). All the four items loaded on a common factor, “poor sleep,” with a cumulative variance of 72. 30%. Further, a goodness of fit index (GFI) = 0.994 and weighted root mean square residual (WRMR) = 0.024 of the BSQ supported its unidimensional factor structure.

#### The Severity of Dependence on Khat

SDS-Khat has been found to have a moderate internal consistency, adequate internal homogeneity, convergent validity, and factorial validity in polysubstance-using adults (19). SDS-Khat is a brief measure to assess dependence on khat with five items each scored on the ordinal scale from 0 to 3. The least score of 0 is indicated for a frequency of never to almost never for khat use-related behavior, while a response of 3 indicates a frequency of always or nearly always for khat use-related behavior. Scores for all individual items are added to obtain SDS-Khat total score; a higher score indicates increasing severity of dependence (19). SDS-Khat is a valid and reliable tool for khat-chewing substance users (19). A cutoff score of 6 and above has been used to indicate severe psychological dependence on khat (31).

#### Meta-Cognition Questionnaire

Metacognition is a person's awareness about his own cognitive and emotive abilities (28). A brief measure of metacognition with nine items was developed and validated by Klusmann et al. (28). This structured questionnaire assesses two important aspects of metacognitive ability, namely, metamemory and metaconcentration (20, 28, 32, 33). An adapted version had been found to have adequate psychometric validity in collegiate young adults (32). Each of these nine items is scored on an ordinal scale of 1 (absolutely wrong) to 5 (absolutely true). Metacognition total score (range: 9–45) is obtained by adding scores for all the nine items. Lower scores indicate poor metacognitive ability in the respondent (28, 32). A similar and adapted meta-cognition questionnaire has been found to have robust psychometric validity measures in substance users, university students, and nurses (20, 32, 33).

#### Socio-Demographic Information

Information related to socio-demographic characteristics—age, gender, presence of chronic conditions, presence of chronic conditions in the family, educational status, marital status, monthly income (in Birr), and duration of athletic activity every day (min)—were collected. Self-reported accounts from respondents for the presence of medication of AIDS, cardiovascular complications, diabetes, epilepsy, hypertension, tuberculosis, and any other chronic diseases including mental health issues were recorded.

### Statistical Analysis

All the statistical analysis was performed by SPSS-26.0 version and Factor 10.10.03 for Windows. Participants' characteristics are presented using mean ± SD, range, frequency, and percentage. Binary logistics regression was employed to identify associated factors of poor sleep and related sleep disturbances after verifying the assumptions. Dichotomized measures—(i) presence or absence of poor sleep based on BSQ and (ii) presence or absence of related sleep disturbances—were outcome variables. There were no multivariate outliers as determined by the Mahalanobis criteria: X^2^(10) = 29.59, *p* < 0.001. There was one univariate outlier in the age but was retained after verifying the correctness of the information. There was no issue of multicollinearity and singularity; all predictor variables were significantly correlated with poor sleep except athletic activity: β = −1.71 to 8.7 (unadjusted odds ratio). All predictor variables had linear relation with their log odds; there were no significant *p*-values for the interaction terms between continuous predictor variables and their natural logs in the model. Factor 10.10.03 for Windows was used to perform a factor analysis of the BSQ scores using categorical data assumptions (34, 35).

## Results

### Participants' Characteristics

More than 90% of the polysubstance users participating in this study were men (Table 1). The prevalence of poor sleep, chronic conditions in the participants, and chronic conditions in the participants' family members was 56.4, 60.4, and 61.3%, respectively (Table 1). Most of the polysubstance users (69.9%) were found to have a more severe psychological dependence on khat. The majority of the study population (53.1%) were illiterate or primary-educated. More than half of the polysubstance users (60.4%) in this study were married or stayed with their partners (Table 1). The range of monthly income (in Birr), SDS-Khat total score, meta-cognition total score, and athletic activity every day (min) were 1,500–5,000, 2–12, 16–36, and 0–105, respectively (Table 1). Khat-chewing polysubstance users were the largest group among the polysubstance-using adults (Table 1).

**Table 1:** Participants' characteristics.

**Characteristics**	**Range; mean ±SD/**
**(*N* = 326)**	**frequency (percentage)**
Age	18–43; 27.1 ± 3.7
**Gender**	
Male	294 (90.2)
Female	32 (9.8)
**Poor sleep**	
No	142 (43.6)
Yes	184 (56.4)
**Presence of chronic conditions**	
No	129 (39.6)
Yes	197 (60.4)
**Presence of chronic conditions in the family**	
No	126 (38.7)
Yes	200 (61.3)
**Educational status**	
Illiterate to primary level	173 (53.1)
Secondary to higher level	153 (46.9)
**Marital status**	
Unmarried/divorced/widowed	129 (39.6)
Double/married	197 (60.4)
Monthly income (In Birr)^a^	1,500–5,000; 3,647.8 ± 707.1
SDS-Khat total score	2–12; 6.5 ± 1.3
**Dependence on khat**	
Less psychological dependence	98 (30.1)
More or severe psychological dependence	228 (69.9)
Meta-cognition total score	16–36; 26.6 ± 4.1
Athletic activity everyday (min)	0–105; 15.0 ± 24.4
Polysubstance use with khat chewing	318 (97.5%)^b^
Polysubstance use with alcohol	230 (70.6%)^b^
Polysubstance use with smoking	221 (67.8%)^b^

*SD, standard deviation; SDS-Khat, severity of dependence on khat scale*.

*The presence of chronic conditions was based on self-reported presence of diabetes, hypertension, epilepsy, tuberculosis, AIDS, other cardiovascular complications, and any other chronic diseases. Poor sleep was evaluated by a brief questionnaire; metacognition was assessed by a brief measure developed by Klusmann et al*.

a *One hundred Birr was approximately equal to 2.01 USD on January 23, 2022*.

b *Groups were not exclusive*.

### Associated Factors of Poor Sleep in Polysubstance Users

Associated factors of poor sleep in the polysubstance users are shown in Table 2. A binary regression prediction model was adjusted for age (years) and gender. The prediction model explained 47.8% (Nagelkerke *R*^2^) of the variance in classifying poor sleepers among polysubstance users (36). This model was significant compared to a model with only intercepts as indicated by χ^2^(10, *N* = 326) = 143.58, *p* < 0.001 with 79.4% accuracy in classifying those with poor sleep. The presence of chronic conditions [adjusted odds ratio (AOR = 2.52, 95% confidence interval CI 1.31–4.85), the presence of chronic conditions in the family (AOR = 2.69, 95% CI 1.40–5.20), illiterate to primary level of educational status (AOR = 2.40, 95% CI 1.30–4.04), higher SDS total score (AOR = 1.39, 95% CI 1.13–1.72), and lower meta-cognition total score (AOR = 0.90, 95% CI 0.84–0.97) predicted poor sleep in the polysubstance users (Table 2).

**Table 2:** Logistic regression predicting poor sleep in polysubstance users.

**Predictors**	***p*-value**	**AOR**
		**(95% CI of AOR)**
**Presence of chronic conditions**		
No		1
Yes	<.01	2.52 (1.31–4.85)
**Presence of chronic conditions in the family**		
No		1
Yes	<0.01	2.69 (1.40–5.20)
**Educational status**		
Illiterate to primary level	<0.01	2.40 (1.30–4.44)
Secondary to higher level		1
**Marital status**		
Single/divorced/widowed		1
Double/married	0.09	1.72 (0.93–3.20)
Monthly income	0.10	1.06 (0.98–1.15)
SDS-Khat total score	<0.01	1.39 (1.13–1.72)
Meta-cognition total score	0.01	0.90 (0.84–0.97)
Athletic activity everyday (min)	0.10	1.01 (1.00–1.02)

*Poor sleep was evaluated by a brief structured questionnaire; the presence of chronic conditions was based on self-reported presence of diabetes, hypertension, epilepsy, tuberculosis, AIDS, other cardiovascular complications, and any other chronic diseases. Metacognition was assessed by a brief measure developed by Klusmann et al. Sample size (N = 326)*.

*SDS-Khat, severity of dependence on khat scale; CI, confidence interval; AOR, adjusted odds ratio, adjusted for age (year) and gender*.

### Associated Factors of Poor Sleep-Related Symptoms in Polysubstance Users

Associated factors of poor sleep-related symptoms in the polysubstance users are shown in Table 3. All four models were adjusted for age and gender. Based on the criteria of Nagelkerke *R*^2^, the prediction models explained 54.9, 69.8, 58.0, and 19.3% of the variance in classifying polysubstance users with a subjective report of sleep disturbances, duration of sleep complaints (3 or more months), daytime restlessness, irritability, and tiredness, and report of social and occupational disruptions related to sleep disturbances, respectively.

**Table 3:** Logistic regression predicting poor sleep-related symptoms in polysubstance users.

**Predictors**	**Subjective report of sleep disturbances**	**Duration of sleep complaints**	**Daytime restlessness, irritability, and tiredness**	**Social and occupational disruptions related to sleep disturbances**
	**AOR** **(95% CI of AOR)**	**AOR** **(95% CI of AOR)**	**AOR** **(95% CI of AOR)**	**AOR** **(95% CI of AOR)**
**Presence of chronic conditions**				
No	1	1	1	1
Yes	1.62 (0.80–3.30)	0.98 (0.41–2.38)	1.42 (0.66–3.02)	4.30 (1.47–12.54)^**^
**Presence of chronic conditions in the family**				
No	1	1	1	1
Yes	4.27 (2.10–8.68)^**^	14.40 (5.73–36.20)^**^	5.61 (2.59–12.15)^**^	0.34 (0.11–1.09)
**Educational status**				
Illiterate to primary level	3.30 (1.71–6.36)^**^	2.57 (1.22–5.40)^**^	2.22 (1.06–4.64)^*^	0.36 (0.13–0.97)^*^
Secondary to higher level	1	1	1	1
**Marital status**				
Single/divorced/widowed	1	1	1	1
Double/married	1.17 (0.60–2.29)	3.65 (1.69–7.90)^**^	2.14 (1.05–4.36)^*^	2.20 (0.80–5.99)
Monthly income	0.99 (0.98–1.00)^**^	0.99 (0.98–1.00)	0.99 (0.98–1.00)^**^	0.99 (0.98–1.00)
SDS-Khat total score	1.53 (1.22–1.93)^**^	1.52 (1.18–1.97)^**^	1.87 (1.43–2.43)^**^	0.90 (0.63–1.29)
Meta-cognition total score	0.91 (0.84–0.98)^*^	0.86 (0.78–0.95)^**^	0.85 (0.78–0.92)^**^	0.85 (0.75–0.97)^*^
Athletic activity everyday (min)	1.02 (1.00–1.03)^*^	1.03 (1.02–1.05)^**^	1.00 (0.99–1.02)	0.99 (0.97–1.01)

* *p < 0.05*;

** *p < 0.01; sample size (N = 326)*.

*Poor sleep was evaluated by a brief sleep questionnaire; subjective report of sleep disturbances, duration of sleep complaints (3 or more months), daytime restlessness, irritability, and tiredness; and report of social and occupational disruptions related to sleep disturbances. The presence of chronic conditions was based on self-reported presence of diabetes, hypertension, epilepsy, tuberculosis, AIDS, other cardiovascular complications, and any other chronic diseases*.

*SDS-Khat, severity of dependence on khat scale; CI, confidence interval; AOR, adjusted odds ratio, adjusted for age (year) and gender*.

The model with correlates was significant compared with a model having only intercept χ^2^(10, *N* = 326) = 169.50, *p* < 0.001, which had an accuracy of 80.4% in classifying polysubstance users with a subjective report of sleep disturbances. The model with correlates was significant compared with a model having only intercept χ^2^(10, *N* = 326) = 241.61, *p* < 0.001, which had an accuracy of 87.7% in classifying polysubstance users with 3 or more months of complaints about sleep duration. The model with correlates was significant compared with a model having only intercept χ^2^(10, *N* = 326) = 173.13, *p* < 0.001, which had an accuracy of 83.4% in classifying complaints of daytime restlessness, irritability, and tiredness. The model with correlates was significant when compared with a model having only intercept χ^2^(10, *N* = 326) = 26.74, *p* < 0.01, which had an accuracy of 92.6% in classifying complaints of social and occupational disruptions related to sleep disturbances.

Low metacognition score predicted the presence of all the four sleep complaints: subjective report of sleep disturbances (AOR = 0.91, 95% CI 0.84–0.98), 3 or more months of complaints about sleep duration (AOR = 0.86, 95% CI 0.78–0.95), complaints of daytime restlessness, irritability, and tiredness (AOR = 0.85, 95% CI 0.78–0.92), and complaints of social and occupational disruptions related to sleep disturbances (AOR = 0.85, 95% CI 0.75–0.97; Table 3). Illiterate to primary level of educational status predicted the presence of all the four sleep complaints: subjective report of sleep disturbances (AOR = 3.30, 95% CI 1.71–6.36), 3 or more months of complaints about sleep duration (AOR = 2.57, 95% CI 1.22–5.40), complaints of daytime restlessness, irritability, and tiredness (AOR = 2.22, 95% CI 1.06–4.64), and complaints of social and occupational disruptions related to sleep disturbances (AOR = 0.36, 95% CI 0.13–0.97; Table 3).

A high SDS total score predicted the presence of the three sleep complaints: subjective report of sleep disturbances (AOR = 1.53, 95% CI 1.22–1.93), 3 or more months of complaints about sleep duration (AOR = 1.52, 95% CI 1.18–1.97), and complaints of daytime restlessness, irritability, and tiredness (AOR = 1.87, 95% CI 1.43–2.43; Table 3). The presence of chronic conditions in the family predicted the presence of the three sleep complaints: subjective report of sleep disturbances (AOR = 4.27, 95% CI 2.10–8.68), 3 or more months of complaints about sleep duration (AOR = 14.40, 95% CI 5.73–36.20), and complaints of daytime restlessness, irritability, and tiredness (AOR = 5.61, 95% CI 2.59–12.15; Table 3). The presence of chronic conditions predicted the presence of social and occupational disruptions related to sleep disturbances (AOR = 4.30, 95% CI 1.47–12.54; Table 3).

## Discussion

In the present study, an association was observed between poor sleep and increasing severity of khat dependence, lower metacognition ability, the presence of chronic conditions, the presence of chronic conditions of a family member, and lower educational status. Moreover, a high proportion of polysubstance users had sleep disturbances and severe dependence on khat. Consistent with the crosstalk between substance use and poor sleep, this is the first study to demonstrate further that polysubstance users have sleep complaints such as subjective sleep disturbances, short sleep duration, daytime complaints, and occupational problems related to sleep disturbances.

Mostly, psychostimulants like khat and nicotine are taken along with central nervous system (CNS) depressants like alcohol: either (a) to obtain greater subjective effects when using these drugs together or (b) administration of khat or nicotine may offset alcohol's depressant effects. Alcohol may temper khat and nicotine-induced stimulants effects like anxiety and impulsive behavior (37, 38). Given that the half-life of cathinone is 1.5 h (39), this combination may be worse, as the effects of khat may wear off quicker than alcohol (half-life is 4–5 h), triggering individuals to consume khat repeatedly. This may lead to a fatal inhibited state in these individuals when the effects of alcohol are felt in isolation. Indeed, polysubstance users have poor health outcomes than single-drug users (38). As such, the present study revealed that PSU of khat, alcohol, and nicotine significantly correlated with poor sleep, poor sleep-related disturbances, and metacognitive deficits.

This study showed that among polysubstance users, the majority of users chewed khat (97.5%), and ~56% of the total population showed poor sleep. This PSU behavior among khat users is similar to the findings of prior studies, which also reported that khat users often use other substances (19, 40). Neurobiological mechanisms of khat which mimic amphetamine-like effects on the sleep circadian system are just beginning to be understood. Dopamine is an important neurotransmitter that modulates the reward circuitry and regulates alertness, thus implicated in the sleep–wake cycle. It has been demonstrated that repeated use of amphetamine-like drugs led to increased sleep latency and a decrease in the total sleep time and slow-wave and rapid eye movement (REM) sleep (41). Sleep changes may further downregulate dopamine receptors, thereby increasing vulnerability to drug misuse (42). Indeed, PSU may elevate the risk of comorbid psychopathology and cognitive dysfunction (13). In support, we and others have previously demonstrated that concurrent use of khat and tobacco smoking (43) and concurrent use of alcohol, khat, and tobacco smoking were associated with poor sleep (18). In animal models of behavioral sensitization, which is a model of drug addiction, nicotine potentiated the amphetamine behavioral response and dopaminergic efflux in rats (44); in the drug reinforcement animal model, nicotine enhanced incentive motivational effects of amphetamine (45). In the light of the dynamic interaction effects between nicotine, amphetamine, and alcohol on hypocretin/orexin, GABAergic, dopaminergic, and cholinergic neurotransmission, it is imperative to systematically study the neurological comorbidities in polydrug users to reveal new insights into the management of these addicts (41).

The present study also identified poor sleep-related disturbances in polysubstance users with the binary logistic regression models that were significantly associated with a subjective report of sleep disturbances and three and more months of sleep complaints. These findings are consistent with a previous study, which demonstrated that adolescents' substance use is significantly associated with sleep disturbances, especially in maintaining sleep regularity, timing, efficiency, and duration (46). Recent evidence support changes in the gene expression level of *Per2* with heavy drinking in alcoholics (47). These changes are modulated by the dopaminergic transmission between the ventral tegmental area and nucleus accumbens involving melatonin hormone, levels of which promote circadian rhythm, or via gene expression by the CLOCK protein (48, 49). Moreover, drugs of abuse mediate direct changes in the circadian rhythm independent of suprachiasmatic nucleus (SCN) or light/dark cycle (50, 51) and also changes the sensitivity of various drugs of abuse like opiates, nicotine, stimulants, and alcohol based on the diurnal cycle (51).

As most study participants were khat users, it is possible to speculate that repeated use of khat, alcohol, and tobacco may have adverse consequences on attention and psychomotor function. Chronic amphetamine use promoted deficits in psychomotor functioning, attention, and sleep disruption (52, 53). Moreover, the concurrent use of nicotine and amphetamine in female rats demonstrated potentiation of behavioral response with an increase in the dopamine concentrations in the striatal slices rich in dopaminergic receptors (44).

Alcohol, on the other hand, is a cytochrome p450 inhibitor; chronic alcohol use may downregulate GABAergic receptors and stimulate the glutamatergic receptors, thereby leading to hyperarousal impulsive behavior state and further increase vulnerability to affective and substance use disorders (54, 55). Sleep disorders may confer vulnerability to future neuropsychiatric complications and cognitive dysfunction (56). Concurrent use of alcohol and methamphetamine (METH) showed impairment of learning and memory compared to METH alone in rats (57). Additional evidence in rats demonstrated that the use of alcohol and METH produced synergy to impair hippocampal-mediated spatial memory compared to using METH alone (58). Alcohol administration alone did not impair the spatial memory, suggesting synergistic effects when these drugs were combined (58). Furthermore, when rat hippocampus was assessed for markers of oxidative stress, the use of alcohol and METH showed a synergistic increase in reactive oxygen species, which are detrimental for the cell compared to the use of either drug alone. Hence, concurrent use of drugs may cause the drugs to interact with each other and may have additive or synergistic effects on the physiological system.

Consistent with the predisposition to sleep changes, cognitive deficits may also interplay, albeit concurrently, following substance use (59, 60). In the present study lower metacognition score predicted the presence of all four sleep complaints in polydrug users. This is in line with previous preclinical and clinical neuroimaging studies, which showed that differential substance users have differential changes in the gray matter volume that are associated with psychomotor, affective, and cognitive deficits (22, 61–64). Furthermore, long-term use of drugs with abuse potential may produce excitotoxicity by over-activation of glutamatergic and dopaminergic synapses, thus leading to neuronal damage and associated behavioral changes (65–67).

In the present study increasing severity of khat dependence was significantly associated with sleep complaints, namely, subjective reports of sleep disturbances, sleep duration complaints (3 or more months), daytime restlessness, irritability, and tiredness. This is in line with other studies, which demonstrated that drug users struggle with internalizing and externalizing problems including difficulty in sleep maintenance, sleep efficiency, and sleep duration (46, 68). Family social support buffers the effects of internalizing problems observed in substance users. Therefore, family involvement may moderate the risk factors associated with substance use and related emotional and behavioral challenges (69). However, if family members of substance users have chronic conditions, then they are less likely to buffer the effects of internalizing problems. This possibly explains the relationship of chronic conditions in family members with various sleep complaints and overall poor sleep because family members with chronic conditions may be less likely to constructively contribute to moderate the effects of internalizing problems (69).

## Limitations of the Study

The current study's results must be viewed in light of the study's cross-sectional nature. Also, the present study only focused on poor sleep and associated sleep disturbances and metacognition. Other factors that may impact sleep were not considered. The collection of data was done by using a validated questionnaire, which lacked objective sleep metrics. Also, illicit drug users were questioned about their drug use (duration in months) with a recall period of 1 month which may be associated with recall bias. Future studies should explore longitudinal study design in a cohort group of individuals who may use CNS stimulants and depressants together and compare to single-drug users or normal subjects. Nevertheless, our study is the first to report a high prevalence of sleep complaints and their association with dependence, metacognitive deficit, and socio-demographic factors in polysubstance users. From the data presented in this study, it is not possible to discern differences in associated factors of poor sleep/poor sleep-related symptoms in discrete groups of polysubstance users. Future studies with case–control designs involving distinct groups of polysubstance users with longitudinal data collection may help in identifying relationships between sub-groups of polysubstance users and sleep.

## Conclusion

This study demonstrates that poor sleep, khat dependence, and metacognitive deficits are highly prevalent in community polysubstance users. Moreover, poor sleep is associated with higher khat dependence, lower metacognitive ability, lower educational status, and the presence of chronic conditions in polysubstance users or their families.

## Data Availability Statement

The raw data supporting the conclusions of this article will be made available by the authors, without undue reservation.

## Ethics Statement

The studies involving human participants were reviewed and approved by Human Institutional Ethics Committee, College of Health Sciences, Mizan-Tepi University, Mizan-Aman, Ethiopia. The patients/participants provided their written informed consent to participate in this study.

## Author Contributions

MM, MS, HH, AMA, DN, ET, SS, SRP, and AB conceptualized the study and its methodology and were involved in data collection and curation also. MM and MK did the data analysis and wrote and edited the manuscript. AHA, MS, HH, AMA, and AB were involved in supervision. All authors reviewed and approved the manuscript.

## Funding

Researchers Supporting Project number (RSP-2021/382), King Saud University, Riyadh, Saudi Arabia. The authors extend their appreciation to the Deanship of Scientific Research at Majmaah University for funding this work under Project Number (R-2022-113).

## Conflict of Interest

SRP is employed by Somnogen Canada Inc. The remaining authors declare that the research was conducted in the absence of any commercial or financial relationships that could be construed as a potential conflict of interest.

## Publisher's Note

All claims expressed in this article are solely those of the authors and do not necessarily represent those of their affiliated organizations, or those of the publisher, the editors and the reviewers. Any product that may be evaluated in this article, or claim that may be made by its manufacturer, is not guaranteed or endorsed by the publisher.

## Abbreviations

SDS-Khat: severity of dependence on khat
PSU: polysubstance use
BSQ: brief sleep questionnaire.
